# Supplementary material for: Comparative multiomics analysis of cell physiological state after culture in a basket bioreactor
Source: Sci Rep. 2022 Nov 23;12:20161. doi: 10.1038/s41598-022-24687-4 (PMC9686226; doi:10.1038/s41598-022-24687-4)
Supplement: Supplementary file 1 — Supplementary Information 1. [file 41598_2022_24687_MOESM1_ESM.zip › raw data/Transcriptomic raw data/6.Enrichment/2.KEGG/TE1vsTC1/DOWN/src/csab05200.html]

csab05200


- csab:103223483

- Down regulated genes

103223483(-3.35312742084333)
- csab:103224207

- Down regulated genes

103224207(-1.32692055344416)

- csab:103224900

- Down regulated genes

103224900(-1.42998371610984)

- csab:103246479

- Down regulated genes

103246479(-1.5490263767958)

- csab:103230197

- Down regulated genes

103230197(-2.07431149272152)

- csab:103220342

- Down regulated genes

103220342(-2.50672933639729)

- csab:103223483

- Down regulated genes

103223483(-3.35312742084333)
- csab:103224207

- Down regulated genes

103224207(-1.32692055344416)

- csab:103218917

- Down regulated genes

103218917(-2.19478759342619)

- csab:103239621

- Down regulated genes

103239621(-1.32776691017615)

- csab:103237005

- Down regulated genes

103237005(-1.92426444229972)

- csab:103223920

- Down regulated genes

103223920(-1.66241974207187)

Close
